# Nutritional composition of ultra-processed plant-based foods in the out-of-home environment: a multi-country survey with plant-based burgers

**DOI:** 10.1017/S0007114524000023

**Published:** 2024-05-28

**Authors:** R. E. Vellinga, H. L. Rippin, B. G. Gonzales, E. H. M. Temme, C. Farrand, A. Halloran, B. Clough, K. Wickramasinghe, M. Santos, T. Fontes, M. J. Pires, A. C. Nascimento, S. Santiago, H. E. Burt, M. K. Brown, K. H. Jenner, R. Alessandrini, A. M. Marczak, R. Flore, Y. Sun, C. Motta

**Affiliations:** 1 Centre for Nutrition, Prevention and Health Services, National Institute for Public Health and the Environment (RIVM), Bilthoven, The Netherlands; 2 Special Initiative on NCDs and Innovation, WHO Regional Office for Europe, Copenhagen, Denmark; 3 Department of Public Health and Primary Care, Faculty of Medicine and Health Sciences, Ghent University, Gent, Belgium; 4 Food and Nutrition Department, National Institute of Health Doutor Ricardo Jorge (INSA), Lisbon, Portugal; 5 Wolfson Institute of Population Health, Barts and The London School of Medicine and Dentistry, Queen Mary University of London, London, UK; 6 Obesity Health Alliance, Wells Lawrence House, London, UK; 7 Physicians Association for Nutrition International, Munich, Germany; 8 Technical University of Denmark, Kongens Lyngby, Denmark

**Keywords:** Vegan burgers, Plant-based foods, Ultra-processed foods, Out-of-home, Food environment

## Abstract

Ultra-processed plant-based foods, such as plant-based burgers, have gained in popularity. Particularly in the out-of-home (OOH) environment, evidence regarding their nutritional profile and environmental sustainability is still evolving. Plant-based burgers available at selected OOH sites were randomly sampled in Amsterdam, Copenhagen, Lisbon and London. Plant-based burgers (patty, bread and condiment) (*n* 41) were lab analysed for their energy, macronutrients, amino acids and minerals content per 100 g and serving and were compared with reference values. For the plant-based burgers, the median values per 100 g were 234 kcal, 20·8 g carbohydrates, 3·5 g dietary fibre and 12·0 g fat, including 0·08 g TFS and 2·2 g SFA. Protein content was 8·9 g/100 g, with low protein quality according to amino acid composition. Median Na content was 389 mg/100 g, equivalent to 1 g salt. Compared with references, the median serving provided 31% of energy intake based on a 2000 kcal per day and contributed to carbohydrates (17–28%), dietary fibre (42%), protein (40%), total fat (48%), SFA (26%) and Na (54%). One serving provided 15–23% of the reference values for Ca, K and Mg, while higher contributions were found for Zn, Mn, P and Fe (30–67%). The ultra-processed plant-based burgers provide protein, dietary fibre and essential minerals and contain relatively high levels of energy, Na and total fats. The amino acid composition indicated low protein quality. The multifaceted nutritional profile of plant-based burgers highlights the need for manufacturers to implement improvements to better support healthy dietary habits, including reducing energy, Na and total fats.

Global meat production has more than doubled since 1961^(1)^ and so have the environmental impacts^(2)^. The trend to move from an animal-based diet towards a more plant-based diet is a key component of initiatives supporting both healthier eating and environmental sustainability^(3,4)^. There is a large body of evidence concluding that limiting the consumption of animal-based foods may lower environmental pressure^(4–6)^.

A shift towards plant-based diets has the potential to also facilitate a decrease in non-communicable diseases (NCD). The rise in NCD is a growing part of the disease burden in Europe and the leading cause of morbidity and mortality in the WHO European Region^(7,8)^. Additionally, the growing burden of overweight and obesity in the European Region, itself both an NCD and a risk factor for other NCD, is a continued public health challenge. In the WHO European Region, overweight and obesity affect almost 60 % of adults and nearly one in three children (29 % of boys and 27 % of girls)^(9)^.

Research shows that compared with animal-based foods, plant-based foods are lower in total energy and are sources of antioxidants, fibre and other essential nutrients^(3)^. Studies have found that predominantly vegetarian and vegan populations with no or a low intake of animal-based foods have lower prevalence rates of overweight and obesity^(3,10)^. In addition, studies have found that high amounts of red and processed meat consumption (i.e. ≥ 100–120 g and 50 g per day, respectively) are associated with a 10–20 % greater likelihood of developing cancer, type 2 diabetes, stroke, coronary heart disease and heart failure^(11,12)^.

The transition towards more plant-based diets has stimulated the food industry to develop new plant-based foods and has coincided with expanding markets^(13)^. While not all, many of the these new industrially developed foods can be classified as ultra-processed foods^(14)^. For instance, approximately 80 % of plant-based burger patties evaluated in major Australian supermarkets were categorised as ultra-processed foods^(15)^. With a greater number of plant-based foods being developed and made available, including ultra-processed, quick and affordable foods, there is a need to know how the nutritional profile of these products affects diet quality and subsequently NCD^(13,16)^. A number of studies assessed the nutritional composition of plant-based foods based on nutrition information provided on label^(17–19)^, hence evidence from the out-of-home (OOH) environment is lacking.

In recent years, there has been a rapid increase in the use of digital food environments, the online settings through which flows of services and information that influence people’s food and nutrition choices and behaviour are directed^(20,21)^. As a result, there has been increased demand for food in the OOH environment, particularly for food ordered through meal delivery apps^(20)^, where ultra-processed convenience foods, including plant-based products, dominate. With a lack of data on the nutritional content of food in the OOH environment due to different regulations regarding nutritional labelling compared with retail products, it is necessary to gather nutrition information on these foods to allow consumers to make healthier and sustainable informed choices^(22)^.

To help build a nutrient profile for the proliferation of ultra-processed plant-based foods in the OOH environment, this study focuses on plant-based burgers as a key example. Laboratory analyses were conducted to gather information on the nutrient content of plant-based burgers in selected cities across the WHO European Region. This multi-country survey provides evidence to initiate the building of an evidence base on which informed policy decisions can be made to improve population health whilst safeguarding the health of the planet.

## Methods

Cities in four WHO European Member States were selected for the study in a convenience sample that covers the breadth of the region: Amsterdam, Copenhagen, Lisbon and London. As this is a small-scale study to initiate the building of a wider evidence base, only a limited number of cities were identified.

### Mapping the sample sites

Representatives from each selected city were asked to determine the location and number of OOH sites that offered plant-based burgers through an online search via Google, TripAdvisor or other related websites. This was done using both English and a local translation of the defined search terms such as ‘vegan *or plant-based burger + name of the city’ and ‘vegan *or plant-based restaurant + name of the city’. Multi-national and country-specific food delivery websites including Deliveroo, Uber Eats and Take-away were also used to search for plant-based burgers. The results from this search were cross-checked against the online search engine list. Personal referrals by country representatives were used to complete the list. A final list of locations of the sampling sites for each city was plotted using a Google My Map maps.

City-specific sampling strategies were used to understand the number and density of OOH sites in each city. For Amsterdam, Copenhagen and Lisbon, sites were classified according to neighbourhood, and from each area, a sample of ten was drawn (eleven for Amsterdam). In London, the city centre (London Underground zone 1) was sampled and was accordingly classified into four areas (North, East, South and West). To achieve the target sample size of ten plant-based burgers per city, the number of burgers purchased within each area was determined by dividing the number of sites in the particular area to the number of total sites in the city and then multiplying by ten. The OOH plant-based burger sites were then selected by random sampling with an Excel function (=RANDBETWEEN()) for Amsterdam, Copenhagen and Lisbon, and randomizer.org was used for London.

### Data collection

A representative from each country visited the identified sites in person and physically purchased the plant-based burger samples. A sample equivalent to one serving ‘as sold’ was procured from each site identified in the mapping exercise. If a burger could not be purchased, for instance because sites were closed, the list derived from the mapping exercise was consulted and the next available site was chosen. Samples were collected ‘as sold’ and included a patty and bun element and may also have included other plant-based components such as plant-based cheese, sauces and condiments, if this was how the product was sold. Samples did not include any side dishes such as fries, chips and crisps and no extra options such as extra plant-based cheese and extra sauce if the consumer had to specifically request these items. If a site had more than one burger option, the best-selling burger was chosen; this was determined by the representative from each country, e.g. by asking the server or by checking popularity on food delivery websites/apps. Each sample was labelled with a reference number, the name of the plant-based burger, the name and full address of the sampling site and the date of sampling. The menu item name and ingredient list or description of each sample were recorded on collection In order to minimise bias, the collection of samples at each location was carried out on the same day. If a site was closed on the day of data collection, it was not included in the study, and an alternative site was chosen as described above.

All samples were placed in zip lock bags and labelled with a reference number. Samples were stored at −20°C freezer until delivery to the laboratory in Lisbon, Portugal. Delivery was via courier with a certified −20°C cold chain.

### Nutritional assessment/lab analysis

Laboratory analysis to determine the nutritional composition was performed at Instituto Nacional de Saúde Doutor Ricardo Jorge (INSA), Lisbon, Portugal. Upon arrival in Lisbon, each sample was unpacked, weighed, homogenised, aliquoted and frozen as soon as possible until laboratory analyses could be undertaken. For proximate analysis, samples were analysed for moisture, total protein, fat, carbohydrates including sugars and total dietary fibre contents. The fatty acid profile, including saturated and *trans*-fatty acids, Na and minerals, and amino acid composition were also determined. Proximate and mineral analysis were performed according to the methods described by Nascimento et al. (2014)^(23)^. Moisture and ash contents were determined by gravimetric methods using a dry air oven and a mufﬂe furnace, respectively. Quantiﬁcation of total fat was performed after an acid hydrolysis method followed by a Soxhlet extraction (Foss Soxtec). Quantiﬁcation of total protein was determined by the Kjeldahl method (Foss Kjeltec). The content of total dietary ﬁbre was determined using an enzymatic–gravimetric method, with heat stable α-amylase, protease and amyloglucosidase as enzymes for digestion (Merck). Minerals were determined after acid digestion with nitric acid, followed by an inductively coupled plasma optical emission spectrometer analysis (ICP-OES Thermo iCAP 6000 series). Fatty acid proﬁle was determined using a gas chromatographer (Agilent 6890N Network GC System), equipped with a ﬂame ionisation detector and according to the ISO 12 966 (2015–2017) and the Commission Regulation (EC) No. 796/2002 (2002), with modiﬁcations, as described by Albuquerque et al. (2016)^(24)^. The amino acid profile was analysed using liquid chromatography (Acquity UPLC, Waters, USA), equipped with a photodiode array detector after acid hydrolysis and a pre-derivatisation as described by Motta et al. (2016)^(25)^.

### Data analysis

Descriptive data on the burgers were summarised and presented as median interquartile rage (IQR), 5th and 95th percentile. Outcomes are presented for the entire sample and include energy, macronutrients and minerals per 100 g and per serving size. Outcomes per serving size were compared with reference values for healthy men and women aged ≥ 18 years (online Supplementary Table 1) derived from WHO^(26–30)^ and European Food and Safety Authority (EFSA)^(31–38)^. The energy intake was set at 2000 kcal a day. Protein requirement was calculated based on an average bodyweight of 70 kg. The nutrient values for the median serving burger were compared with reference intakes for macronutrients, and with population reference intakes (PRI) or adequate intakes (AI) if PRI were not available.

Furthermore, descriptive data were used to summarise amino acid composition of the plant-based burgers. Amino acid scores reflect the amount of an amino acid relative to the reference amount of that amino acid per gram of protein. Scores were calculated using the essential amino acids histidine (His), isoleucine (Ile), leucine (Leu), lysine (Lys), sulphur amino acids (SAA) (metheonine (Met) and cysteine (Cys)), aromatic amino acids (tyrosine (Tyr), phenylalanine (Phe)), threonine (Thr) and valine (Val), following the formula (amount of amino acids/100 g) divided by (the total amount of protein), divided by (the reference intake for adults), based on WHO report on protein and amino acid requirements (i.e. mg of amino acids per 1 g protein/ mg of amino acids in required pattern)^(39)^. Furthermore, amino acids per serving in the plant-based burgers were compared with daily references^(39)^. For each of the essential amino acids, the relative intake per day was estimated based on the amino acid requirements in mg per day for adults > 18 years with a bodyweight of 70 kg.

## Results

A total of 171 OOH sites selling plant-based burgers were identified in Amsterdam, fifty-nine in Copenhagen, seventy in London and 151 in Lisbon in 2022 between March and May. The locations of these sites were listed and mapped (online Supplementary Figures 1(a)–(d)). The description and characteristics, such as weight, main ingredients, nutritional composition and costs of the plant-based burgers, are shown in online Supplementary File 3. Forty-one plant-based burgers were purchased and analysed. Costs of the purchased burgers varied between €4·50 and €18·00.

Per 100 g the median energy content was 234 kcal (IQR = 50) or 978 KJ (IQR = 205) (Table 1). The median macronutrient composition per 100 g was 20·8 g (IQR = 5·7) carbohydrates, 3·5 g (IQR = 1·8) dietary fibre and 8·9 g (IQR = 3·7) protein. Per 100 g, the burgers contained a median total fat content of 12·0 g (IQR = 4·2), including 0·08 g (IQR = 0·05) TFA, 2·2 g (IQR = 2·3) SFA, 5·2 g (IQR = 3·6) MUFA and 3·3 g (IQR = 1·2) PUFA. The median Na content was 389 mg (IQR = 113) per 100 g, equivalent to 1 g salt.

**Table 1.** Composition of energy, macronutrients and minerals of plant-based burgers per 100 g and per serving

	Per 100 g			Per serving		
	Median	IQR	5th–95th percentile	Median	IQR	5th–95th percentile
Quantity (g)				280·0	65·0	200·3–339·0
Energy (kcal)	233·8	49·6	184·5–295·8	618·8	183·3	469·2–945·8
Energy (kJ)	977·5	204·8	774·5–1231·6	2585·5	763·7	1965·4–3942·6
Carbohydrates (g)	20·8	5·7	17·4–27·4	56·2	17·7	40·4–84·0
Dietary fibre (g)	3·5	1·8	2·0–6·4	10·6	5·9	4·4–18·3
Total fat (g)	12·0	4·2	6·2–19·3	31·9	13·2	16·4–57·3
TFA (g)	0·1	0·1	0·0–0·1	0·2	0·1	0·1–0·5
% TFA /100 g total fat	0·7	0·3	0·3–1·0			
SFA (g)	2·2	2·3	0·9–5·7	5·7	5·6	2·0–19·1
MUFA (g)	5·2	3·6	2·1–10·3	13·7	8·3	5·2–33·9
PUFA (g)	3·3	1·2	1·3–5·9	9·3	4·6	4·3–33·9
Protein (g)	8·9	3·7	5·0–11·8	23·2	9·1	15·7–31·4
Na (mg)	388·9	112·9	246·0–573·5	1086·6	395·6	702·7–1661·6
K (mg)	220·1	85·9	139·8–356·7	607·6	271·1	324·9–1255·0
Mg (mg)	24·8	8·2	14·3–44·6	70·1	33·6	38·1–132·0
Ca (mg)	46·5	34·8	33·0–103·6	125·7	82·3	88·1–337·2
P (mg)	91·3	35·6	66·8–145·7	278·9	93·7	157·8–409·9
Mn (mg)	0·4	0·1	0·3–0·7	1·1	0·4	0·7–2·1
Fe (mg)	1·4	0·5	1·0–2·0	4·0	1·1	2·3–5·8
Zn (mg)	0·9	0·4	0·6–1·3	2·2	1·2	1·3–3·6
Salt (g)	1·0	0·3	1·2–6·8	2·7	1·0	3·4–14·4

TFA, *trans*-fatty acids.

**Fig. 1.**
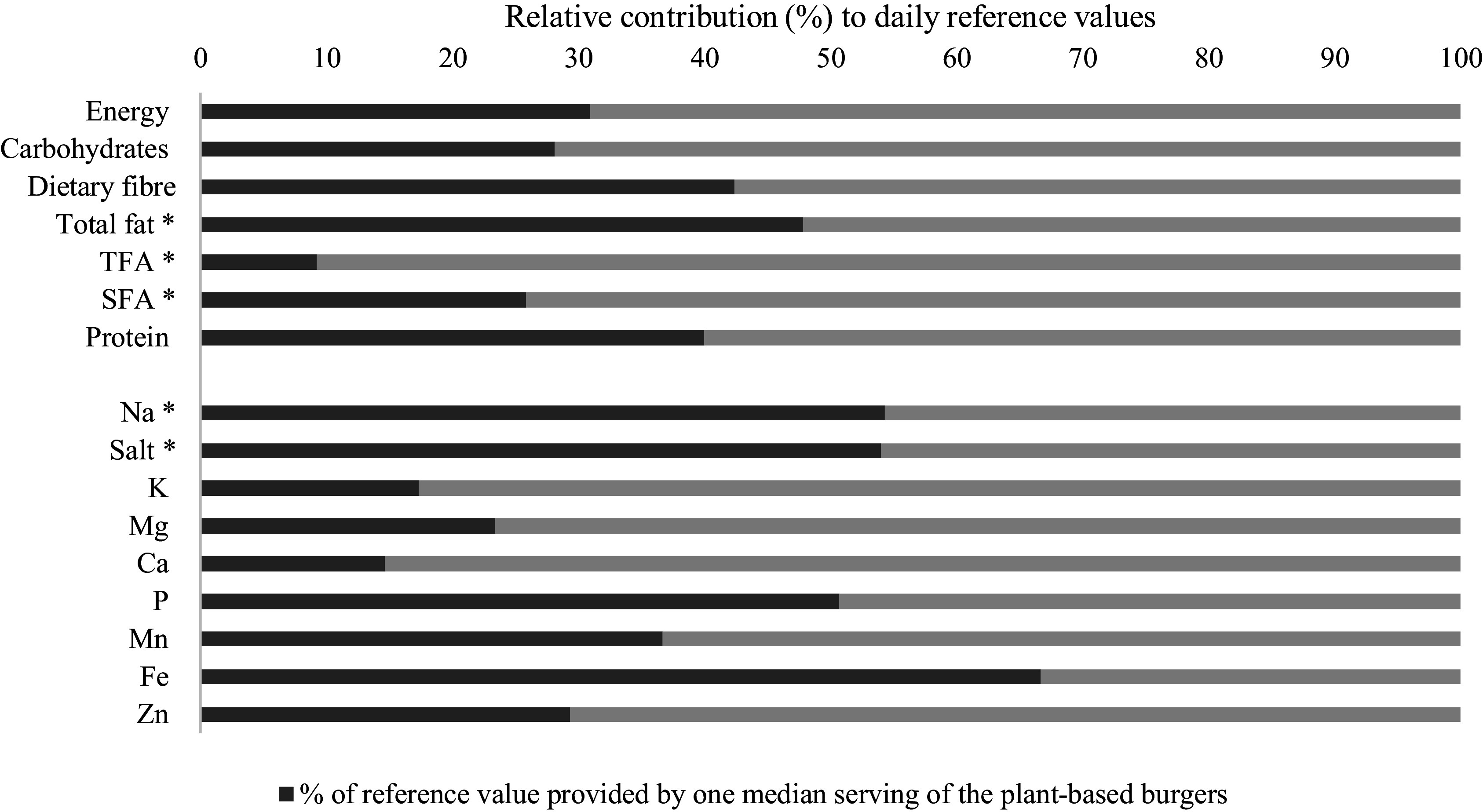
The relative amount of energy, macronutrients and minerals per serving (in %) compared with the daily reference values. *indicates the contribution towards the maximum recommendations.

The median serving size of plant-based burgers was 280 g (IQR = 65), providing 619 kcal (IQR = 183) (Table 1). This accounts for 31 % of energy intake, based on a 2000 kcal per day diet (Fig. 1). One median serving provided 56·2 g (IQR = 17·7) carbohydrates, accounting for 17–28 % of the reference values. One median serving provided 10·6 g (IQR = 5·9) dietary fibre and 23·2 g (IQR = 9·1) total protein, corresponding to, respectively, 42 % and of 40 % of reference values for dietary fibre (25 g) and the protein (58·1 g) (PRI). The median amount of total fat per serving was 31·9 g (IQR = 13·2), equating to 48 % of the maximum level. The fatty acid composition per median serving of plant-based burgers included 0·2 g TFA, 5·7 g SFA, 13·7 g MUFA and 9·3 g PUFA. One median serving accounted for, respectively, 9 % and 26 % of the daily maximum levels for TFA and SFA. The median Na content per serving was 1086·6 mg (IQR = 395·6), equivalent to 2·7 g salt, and 54 % of the daily maximum level. One median serving of plant-based burgers provided 15 % of the reference value for Ca (AI), and, respectively, 17 % and 23 % of the reference values for potassium (PRI) and Mg (AI). Contributions to the reference values for Zn (30 % of PRI), Mn (38 % of AI), phosphorus (51 % of AI) and Fe (67 % of PRI) were higher.

**Fig. 2.**
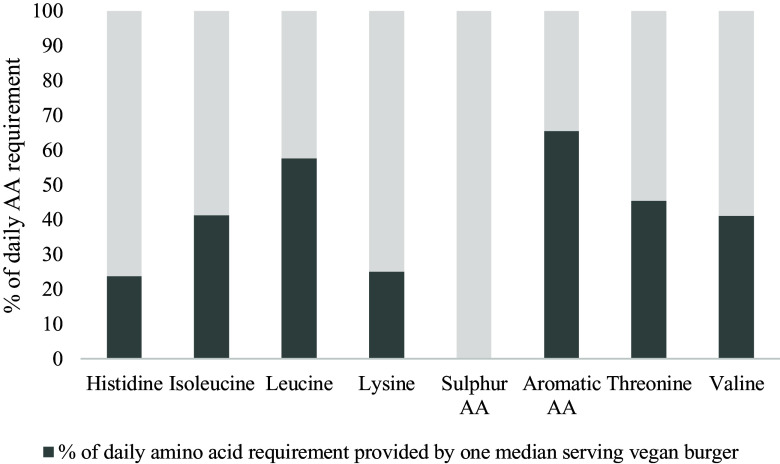
The relative amount of amino acids per serving (in %) compared with the daily reference values. Aromatic amino acids, tyrosine and phenylalanine; sulphur amino acids, meteonine and cysteine.

Median amino acid scores (AAS) varied between 0 for SAA (Met and Cys) and 43 for His to 110 for Leu and 127 for aromatic amino acids (Tyr and Phe) (online Supplementary Table 2). The amino acid composition of the plant-based burgers indicates low protein quality. The (median) relative contribution towards the daily recommendations for essential amino acids were 0 % for SAA, 24 % for His, 25 % for Lys, 41 % for Ile, 41 % for Val, 45 % for Thr, 58 % for Leu and 65 % for aromatic amino acids (Fig. 2).

## Discussion

Ultra-processed plant-based foods have gained in popularity as a perceived healthier and more sustainable alternative to animal-based foods, yet the evidence regarding their nutritional profile, environmental sustainability and impact on NCD is still evolving^(13)^. This study aimed to contribute to the understanding of the nutrient profile of ultra-processed plant-based foods in the OOH environment, by focusing on plant-based burgers. The study provides an overview of the nutritional content and amino acid composition of plant-based burgers available in OOH environments in Amsterdam, Copenhagen, Lisbon and London. Our results indicate that while plant-based burgers are a source of (low quality) protein, dietary fibre and essential minerals, they also contain relatively high levels of energy, Na, total fat and SFA, which are directly linked to NCD.

Our study findings are consistent with existing literature indicating that ultra-processed plant-based foods such as plant-based burgers can provide dietary fibre, (low-quality) plant-based protein and minerals^(40–43)^. Therefore, their inclusion in the diet may contribute to meeting daily requirements and may have lower environmental impacts than meat-based burgers. Additionally, the intake of plant-based protein, dietary fibre and minerals, which are abundantly present in plant-based burgers, has been linked to a reduced risk of certain NCD such as CVD^(26,30,44)^. While ultra-processed plant-based foods can serve as a source of certain nutrients, the extent to which these foods contribute to overall nutrient intake is influenced by various factors, including but not limited to an individual’s dietary pattern, their nutritional status and the bioavailability of the nutrients in question. The magnitude of the contribution made by the consumption of the burgers to daily nutrient intake may vary depending on dietary patterns of individuals. This is beyond the scope of the current study. Nevertheless, it has been reported that current intake levels of certain essential nutrients, including dietary fibre^(45)^, and minerals, such as Fe^(46)^ and potassium^(26)^, are, in general, below the daily recommendations in Europe. Therefore, the consumption of these burgers may contribute to daily requirements, independent of the consumption of other foods.

On the other hand, in agreement with prior research, the plant-based burgers are energy-dense and contain relatively high amounts of added salt and fat, which can adversely impact their overall healthfulness^(19,41–43)^. In the WHO European Region, energy, sugar, fatty acids and salt intakes generally exceed the recommended levels and for health reasons their intake should be decreased^(30)^. For instance, a high intake of Na has been associated with an increased risk of NCD such as CVD, stroke and high blood pressure^(27,30)^. Similarly, the consumption of excessive sugar and unhealthy fatty acids has been linked to a heightened risk of obesity, type 2 diabetes and other NCD^(28,30,47)^. Therefore, besides the beneficial nutritional factors present in ultra-processed plant-based foods, they are also a source of unhealthy compounds. This contradiction raises the question whether the healthier aspects of plant-based burgers outweigh the less healthy aspects, which is contingent on an individual’s dietary patterns and nutritional status. Factors such as the frequency and quantity of burger consumption, as well as the overall dietary context in which burgers are consumed, can affect the potential health outcomes of their consumption.

The AAS of the plant-based burgers analysed in our study ranged from 0 for SAA to 127 for aromatic amino acids, indicating low protein quality. AAS < 100 indicate less than the recommended amino acids per 1 g protein, while AAS above 100 indicate sufficient of the recommended amino acids per 1 g protein^(39)^. To synthesise a protein from amino acids, a specific quantity of amino acids is required. The amino acid that exists in the lowest quantity becomes the limiting factor, and the protein cannot be constructed beyond this particular amino acid’s availability. Although Lys is often the limiting factor, in our study, Cys and Met were the limiting amino acids as they were below the limit of detection^(25)^. Sulphur-containing amino acids can be destroyed depending on the cooking procedures, especially in foods from vegetable sources. Cooked pulses and meat substitutes are the foods that contribute less to the recommended intake on SAA (Cys and Met)^(48)^. Nevertheless, in order to predict protein quality, it is imperative to incorporate digestibility factors. The quality of protein can be predicted by comparing the pattern of digestible amino acid composition with human amino acid requirements: the digestible indispensable amino acid score^(39)^. Furthermore, the amino acid bioavailability in plant-based foods may differ from animal-based foods^(48)^. At last, if complementary foods are consumed within 3–4 h, deficient amino acids can be supplied, enhancing the amino acid content.

Additionally, as for amino acids, it is important to consider the potential impact of additives and nutritional factors present in plant-based foods. The inclusions of a variety of additives to intimidate the sensory properties of meat have raised concerns about the nutritional and food safety aspects of ultra-processed plant-based foods^(48)^. Factors (such as phytates) affecting the bioavailability of the nutrients in the burgers^(43)^ and may, for instance, inhibit the absorption of certain nutrients and therefore influence their ultimate contribution to overall nutritional status^(43,49)^. The relatively high Fe content of the burgers, for instance, may be largely composed of non-heme Fe, which is primarily found in plant sources and more variable to absorption compared with heme Fe^(49–51)^. Fortification of the burgers cannot be ruled out as it was not within the scope of current study.

Despite the aforementioned considerations, it is possible to compare the burgers to established guidelines that are commonly used to evaluate the nutritional value of foods. In order to encourage or discourage the consumption of certain foods, the WHO Regional Office for Europe has developed the nutrient profile model in 2015 and updated it in 2023^(52)^. This model aims to provide guidance for restricting the marketing of foods to children and classifies foods according to its nutritional composition as whether or not it is nutritionally suitable to be marketed for consumption by children. According to the nutrient profile model, the product category ’Savory plant-based foods/meat analogues’ in which plant-based burgers are situated, marketing is prohibited of plant-based burgers that contain > 17 g fat, > 1 g TFA or > 0·5 g Na per 100 g. In light of the nutritional content of the sampled burgers, including bread and sauces, 10 % of the burgers contained more than the maximum level for total fat, and 20 % of the burgers contained more than the maximum level for Na. Therefore, they exceeded the threshold making them unsuitable to be marketed according to the nutrient profile model^(52)^.

A strength of this multi-country survey lies in its focus on investigating the nutritional content of ultra-processed plant-based foods in various cities across the WHO European Region, which will provide case study evidence to initiate the building of an evidence base on which informed policy decisions can be made to improve population health while safeguarding that of the planet. As there is a rapid increase in the availability of ultra-processed plant-based food in current food environments, this current study highlights the need to critically assess the availability, composition and consumption of those foods in the OOH food environment. Moreover, the nutrient analysis done in this study is a strength since existing studies often used labelling information^(17–19)^. At last, the consideration of plant-based burgers (i.e. patty, bread and condiment) in current study is a major strength as it reflects the nutrients associated with food intake rather than the patty only. For the interpretation of our results, certain limitations should be noted. This study aimed to initiate the building of a wider evidence base for plant-based burgers, but its generalisability is limited by the sample size and coverage of burgers and locations. The study included forty-one plant-based burgers from four cities within the WHO Region Europe (Amsterdam, Copenhagen, Lisbon and London), but these cities might not be representative of other regions. The results might also differ between and within countries, and the samples might not cover all different types of plant-based burgers. However, due to the low sample sizes in each city (*n* 10), no comparison can be made. Furthermore, the current study did not measure micronutrients such as vitamin B_12_, which are mainly present in animal-based foods and important to monitor its adequacy in the transition towards a plant-based diet. Although B_12_ is not naturally present in plant-based foods, the burgers could potentially be fortified with it.

The current multi-country survey provides a case study on ultra-processed plant-based foods, using plant-based burgers as an example. Plant-based burgers have a multifaceted nutritional profile with aspects that support and go against healthy dietary habits. Most of the plant-based burgers did not exceed the maximum levels for total fat, SFA and Na levels according to the WHO nutritional profile model to prevent inappropriate marketing to children marketing^(52)^. In addition, (ultra-processed) plant-based foods often have a ‘health-halo’, being perceived by consumers as healthy^(13,53,54)^, which is not the case necessarily. The food environment, among food marketing and the availability of foods, has a large influence on what consumers unconsciously purchase and consume^(55)^. In general, the marketing of ultra-processed plant-based foods such as plant-based burgers in the OOH environment is strong^(56)^ and, according to our study, they are widely available. Therefore, policy for marketing regulation is needed, and improved awareness of the health and environmental aspects of ultra-processed plant-based foods might be required. Furthermore, the variation in nutrient content between burgers highlights the potential for reformulation of ultra-processed plant-based foods by manufacturers and food handlers and may contribute to more healthier and sustainable plant-based burgers in the OOH environment. Future scaled-up studies on the nutritional composition of ultra-processed plant-based foods are needed and should also be coupled with life-cycle assessments to understand the relative environmental impacts.

### Conclusion

With this study, we provide data to help build an evidence based on which informed policy decisions can be made to improve population health whilst safeguarding the health of the planet. The findings indicate that ultra-processed plant-based foods, such as plant-based burgers, provide protein, dietary fibre and essential minerals, but they also contain relatively high levels of energy, Na and total fats. Despite their potential as a source of protein, the amino acid composition of the plant-based burgers indicated low protein quality. Therefore, ultra-processed plant-based foods in the OOH environment have components that contribute to healthier dietary habits, but also some components are relatively high, which may contribute to increased risk of developing NCD. The multifaceted nutritional profile of plant-based burgers highlights the need for manufacturers to implement improvements to better support healthy dietary habits. These improvements should include reducing energy, Na and total fats.

## Supporting information

Vellinga et al. supplementary material 1 Vellinga et al. supplementary material

Vellinga et al. supplementary material 2 Vellinga et al. supplementary material

## Supplementary material

For supplementary material/s referred to in this article, please visit https://doi.org/10.1017/S0007114524000023
