# Supplementary material for: Nutritional composition of ultra-processed plant-based foods in the out-of-home environment: a multi-country survey with plant-based burgers
Source: Br J Nutr. 2024 Jan 15;131(10):1691–8. doi: 10.1017/S0007114524000023 (PMC11063664; doi:10.1017/S0007114524000023)
Supplement: Vellinga et al. supplementary material 2 — Vellinga et al. supplementary material [file S0007114524000023sup002.docx]

**Supplemental tables and figures**

**Supplemental table 1** – Reference values

**Supplemental table 2** – Composition of amino acids of plant-based burgers per 100g and per serving, and comparison with requirements for essential amino acids.

**Supplemental table 3 –** Overview of sampled burgers and composition **(to Excel)**

**Supplemental figures 1a-1d –** Distribution of out of home sites selling plant-based burgers for Amsterdam, Copenhagen, Lisbon and London.

**Supplemental table 1 -** Reference values.

|  | **Reference value** | **type** | **Source** |
| --- | --- | --- | --- |
| **Energy (kcal)** | The recommended daily energy intake varies based on age, sex, weight, and physical activity level. However, the average recommended daily energy intake for an adult is around 2000-2500 kcal/day. For current study we set energy intake at 2,000 kcal. |  | WHO (30) |
| **Energy (kJ)** | 8368 kJ |  |  |
| **Total carbohydrates (g)** | 45-60 % of total energy | RI | EFSA (31) |
| **Dietary fibre (g)** | 25 g | AI | EFSA (31) |
| **Total fat (g)** | The WHO recommends that total fat intake should not exceed 30% of total energy intake. This means that for an adult consuming 2000 calories per day, the daily fat intake should not exceed 67 grams. | RI | WHO (29) |
| **TFA (g)** | The WHO recommends that TFA intake should be limited to less than 1% of total energy intake. This means that for an adult consuming 2000 calories per day, the daily TFA intake should not exceed 2 grams.  TFA limit is also 2% per 100 g total fat. | RI | WHO (28) |
| **SFA (g)** | The WHO recommends that SFA intake should not exceed 10% of total energy intake. This means that for an adult consuming 2000 calories per day, the daily SFA intake should not exceed 22 grams. | RI | WHO (28) |
| **Protein (g)** | 0,83 g/kw/bw. Based on 0,83g/kg/bodyweight and 70 kg bodyweight, this results in 58.1 g protein | PRI | EFSA (32) |
|  |  |  |  |
| **Na (mg) /Salt (g)** | The WHO recommends that adults should consume less than 5 grams of salt (or 2000 mg of sodium) per day. | Safe and adequate intake | WHO (27) |
| **K (mg)** | The WHO recommends that adults should consume at least 3.51 grams of potassium per day. | PRI | WHO (26) |
| **Mg (mg)** | Male, female 350 mg/day, 300 mg/day* | AI | EFSA (33) |
| **Ca (mg)** | 1000 mg/day (18-24 y)  950 mg/day (≥ 25 y) | PRI | EFSA (34) |
| **P (mg)** | 550 mg/day | AI | EFSA (35) |
| **Mn (mg)** | 3 mg/day | AI | EFSA (37) |
| **Fe (mg)** | 11 mg/day for males  11 mg/day for females* (premenopausalwomen=16; postmenopausal women=11) | PRI | EFSA (36) |
| **Zn (mg)** | Male, female (LPI 300 mg/day) 9.4 mg/day,7.5 mg/day*  Male, female (LPI 600 mg/day) 11.7 mg/day,9.3 mg/day  Male, female (LPI 900 mg/day) 14 mg/day,11 mg/day  Male, female (LPI 1200 mg/day) 16.3 mg/day,12.7 mg/day | PRI | EFSA (38) |
| Abbreviations: AI-= ADEQUATE INTAKE; PRI= POPULATION REFERENCEINTAKE; RI = REFERENCE INTAKE;  *reference value used in current study | | | |

**Supplemental table 2.** Composition of amino acids of plant-based burgers per 100g and per serving, and comparison with requirements for essential amino acids.

|  |  | **per 100 g** | |  | | |  | **per serving** | |  |  |
| --- | --- | --- | --- | --- | --- | --- | --- | --- | --- | --- | --- |
|  | **N** | **Median** | **QRange** | **Mg/g** | **Requirement (mg/g)*** | **Amino acid**  **score*** |  | **Median** | **QRange** | **Requirement per day*** | **% of req** |
| **Histidine(mg)** | 31 | 68 | 39 | 7 | 15 | 43 |  | 167 | 203 | 700 | 24% |
| **Serine(mg)** | 41 | 409 | 192 |  |  |  |  | 1094 | 523 |  |  |
| **Arginine(mg)** | 41 | 281 | 230 |  |  |  |  | 810 | 639 |  |  |
| **Glycine(mg)** | 41 | 300 | 146 |  |  |  |  | 795 | 330 |  |  |
| **Asparagine(mg)** | 41 | 834 | 421 |  |  |  |  | 2273 | 1278 |  |  |
| **Glutamine(mg)** | 41 | 2647 | 1049 |  |  |  |  | 6844 | 2881 |  |  |
| **Threonine(mg)** | 41 | 178 | 106 | 20 | 23 | 87 |  | 477 | 340 | 1050 | 45% |
| **Alanine(mg)** | 41 | 306 | 138 |  |  |  |  | 820 | 413 |  |  |
| **Proline(mg)** | 41 | 622 | 319 |  |  |  |  | 1471 | 884 |  |  |
| **Cysteine(mg)** | 41 | <LoD |  |  |  |  |  |  |  |  |  |
| **Lysine(mg)** | 38 | 239 | 225 | 22 | 45 | 49 |  | 525 | 586 | 2100 | 25% |
| **Tyrosine(mg)** | 41 | 141 | 146 |  |  |  |  | 368 | 364 |  |  |
| **Meteonine(mg)** | 2 | 14 | 8 |  |  | - |  | 0 | 0 |  | - |
| **Valine(mg)** | 41 | 290 | 144 | 30 | 39 | 78 |  | 747 | 487 | 1820 | 41% |
| **Isoleucine(mg)** | 41 | 222 | 149 | 23 | 30 | 77 |  | 578 | 470 | 1400 | 41% |
| **Leucine(mg)** | 41 | 602 | 285 | 65 | 59 | 110 |  | 1573 | 865 | 2730 | 58% |
| **Phenylalanine(mg)** | 41 | 297 | 207 |  |  |  |  | 825 | 510 |  |  |
| **Aromatic amino acids(mg)** | 41 | 436 | 335 | 48 | 38 | 127 |  | 1146 | 860 | 1750 | 65% |
| **Sulphur amino acids (mg)** | 41 | <LoD |  |  | 22 | 0 |  |  |  | 1050 | 0% |
| **Amino Acids Sum(g)** | 41 | 8 | 4 |  |  |  |  | 21 | 10 |  |  |
| *Aromatic amino acids, tyrosine and phenylalanine; sulphur amino acids, meteonine and cysteine; LoD, limitl of detection*  **Calculated as amount of amino acid per 1g protein divided by the reference amount of amino acid per 1g protein (39)* | | | | | | | | | | | |

**Supplemental table 3.** Overview of 41 sampled burgers and composition.

**[external file]**

**Supplemental figures 1a-1d** Distribution of out of home sites selling plant-based burgers for Amsterdam, Copenhagen, Lisbon and London.

| 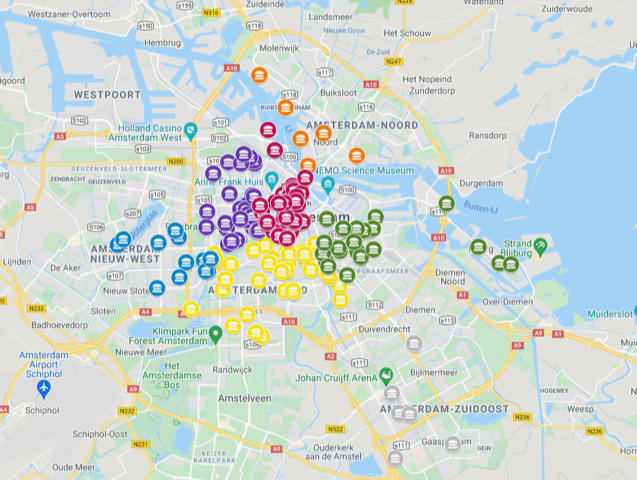  Fig 1a. Distribution of out of home sites, Amsterdam | 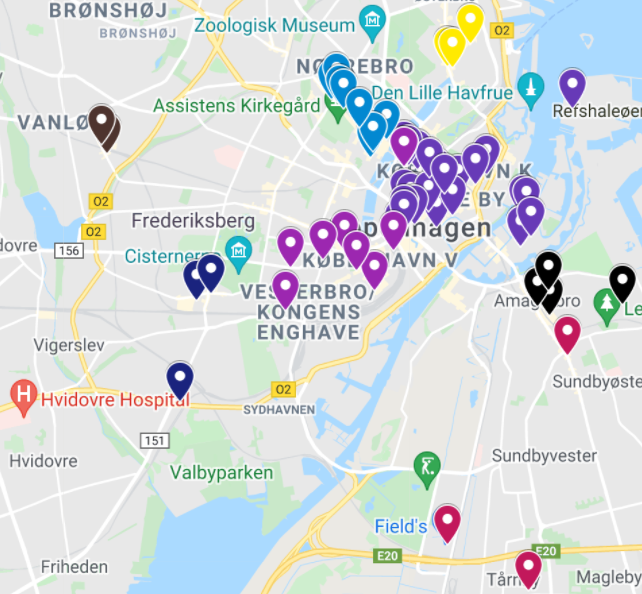  Fig 1b. Distribution of out of home sites, Copenhagen |
| --- | --- |
| 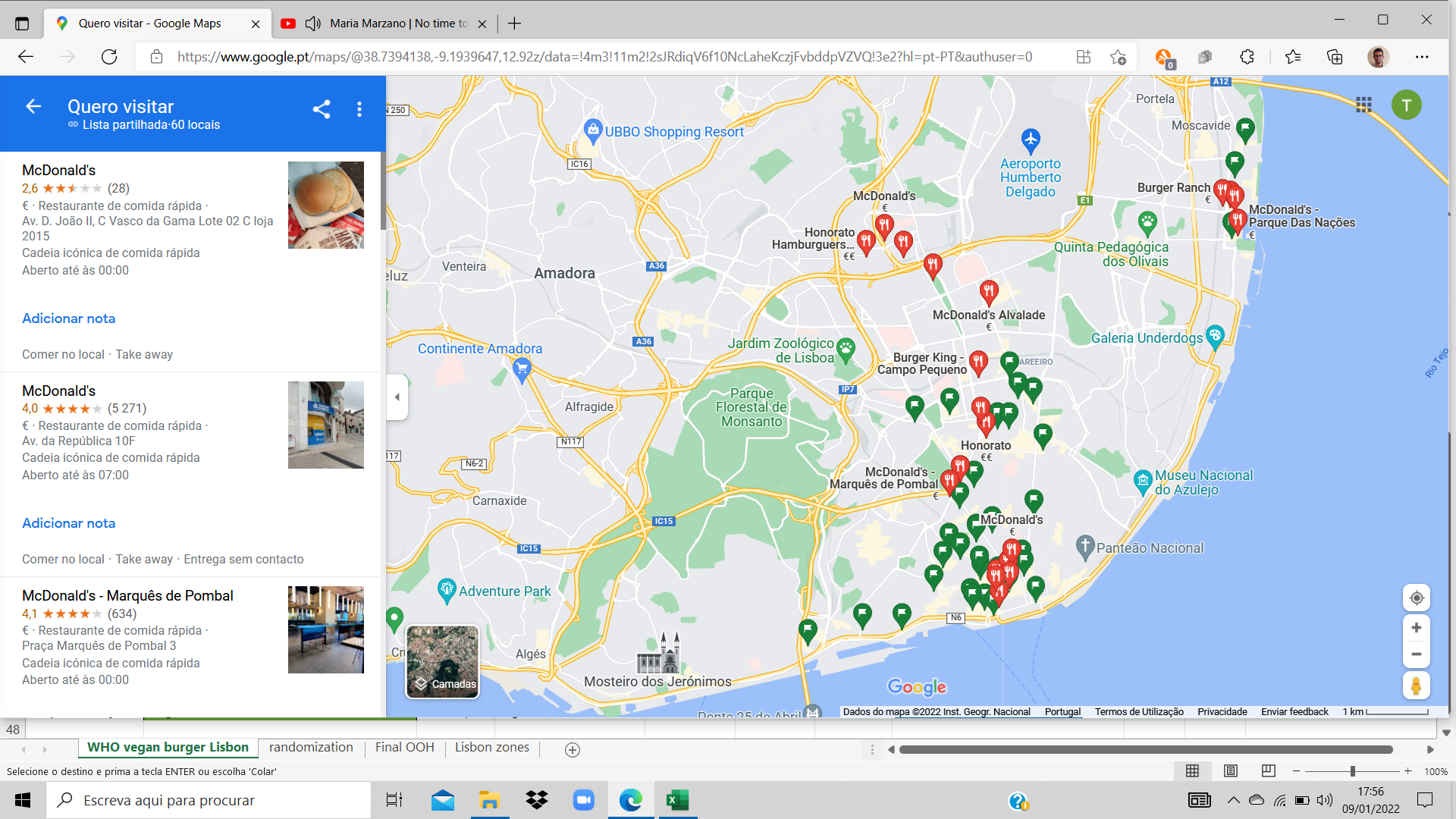  Fig 1c. Distribution of out of home sites, Lisbon | 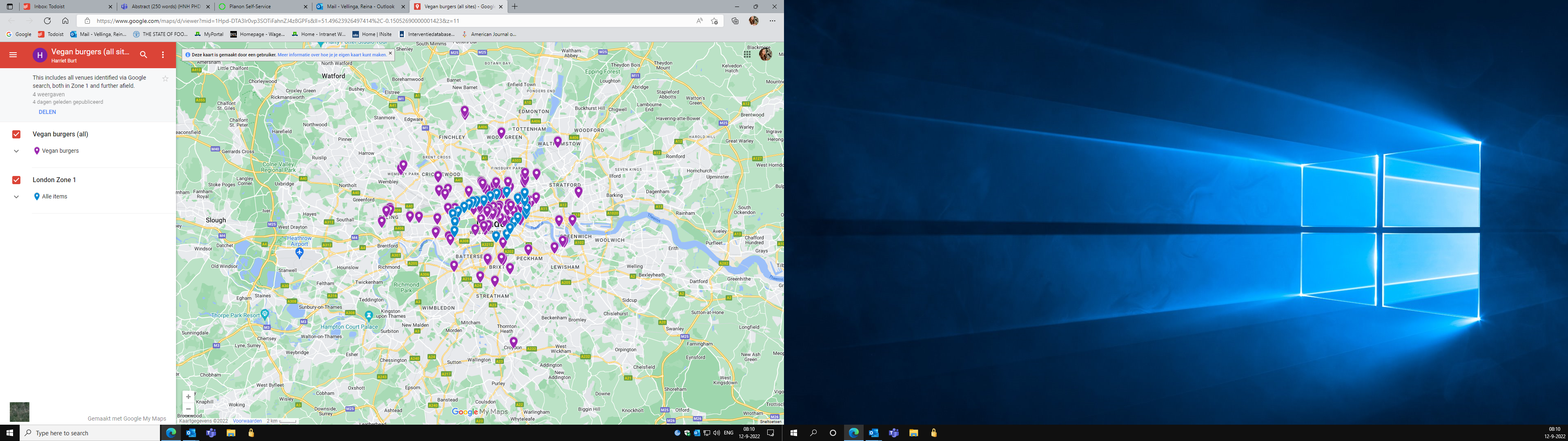  Fig 1d. Distribution of out of home sites, London |
